# Synergistic Effects of Liquid Rubber and Thermoplastic Particles for Toughening Epoxy Resin

**DOI:** 10.3390/polym16192775

**Published:** 2024-09-30

**Authors:** Zhaodi Wang, Yuanchang Lai, Peiwen Xu, Junchi Ma, Yahong Xu, Xin Yang

**Affiliations:** 1 College of Materials Science and Engineering, Nanjing Tech University, Nanjing 211816, China; 201910006628@njtech.edu.cn (Z.W.); 202061203297@njtech.edu.cn (Y.L.); 202261203197@njtech.edu.cn (P.X.); 2 Yangtze River Delta Carbon Fiber and Composites Innovation Center, Changzhou 213000, China; majunchi@ccicyd.com

**Keywords:** resin, liquid rubber, thermoplastic particles, toughening agent, synergistic toughening

## Abstract

This study aims to investigate the toughening effects of rubber and thermoplastic particles on epoxy resin (EP), and to understand the mechanism underlying their synergistic effect. For this purpose, three EP systems were prepared using diglycidyl ether of bisphenol-A (DGEBA) epoxy resin (E-54) and 4,4-Diamino diphenyl methane (Ag-80) as matrix resin, 4,4-diaminodiphenyl sulfone (DDS) as a curing agent, and phenolphthalein poly (aryl ether ketone) particles (PEK-C) and carboxyl-terminated butyl liquid rubber (CTBN) as toughening agents. These systems are classified as an EP/PEK-C toughening system, EP/CTBN toughening system, and EP/PEK-C/CTBN synergistic toughening system. The curing behavior, thermal properties, mechanical properties, and phase structure of the synergistic-toughened EP systems were comprehensively investigated. The results showed that PEK-C did not react with EP, while CTBN reacted with EP to form a flexible block polymer. The impact toughness of EP toughened by PEK-C/CTBN was improved obviously without significantly increasing viscosity or decreasing thermal stability, flexural strength, and modulus, and the synergistic toughening effect was significantly higher than that of the single toughening system. The notable improvement in toughness is believed to be due to the synergistic energy dissipation effect of PEK-C/CTBN.

## 1. Introduction

Epoxy resin (EP) is an essential thermosetting resin due to its good processability, flexible formulation design, and low-volume shrinkage during curing. It has excellent adhesion and mechanical properties, high chemical stability, and low cost [1], making it a popular material in the chemical industry, electronics and electrical appliances, aerospace, transportation, and construction [2,3,4,5]. However, the cured EP molecular chain is a three-dimensional cross-linked network structure, which makes it difficult for the molecular chain to slide. As a result, the cured EP has a high cross-link density and internal stress, which leads to the existence of brittleness, insufficient impact performance, poor fatigue resistance and heat resistance in the cured materials [6,7,8], and limits the application in high-end fields. Therefore, EP must be toughened and modified.

The current EP toughening paths mainly include the following: changing the EP cross-linked network structure to toughen EP; and adding rubber elastomer, thermoplastic resin, liquid crystal polymer, interpenetrating network polymer, hyperbranched polymer, inorganic rigid particles, etc., to EP [1,9]. Among them, the study of the rubber toughened epoxy system was the earliest, which started in the 1960s [10]. The most commonly used toughening material are liquid rubbers, which can be both toughening agents and curing agents. During the curing process of EP, the active groups at both ends of the rubber molecular chain react with the epoxy system to form a three-dimensional network structure. It can effectively improve the inherent brittleness, impact toughness, and elongation of the cured epoxy system [11,12]. The reactive rubbers and elastomers which were used in the epoxy toughening system contain the following: carboxyl-terminated butyl liquid rubber (CTBN), hydroxyl-terminated liquid nitrile rubber (HTBN), polysulfide rubber, and polyurethane rubber [13,14,15]. G Tripathi et al. [16] prepared six toughening components by physically mixing different concentrations of CTBN with EP. The results showed that the tensile strength of the cured materials decreased from 11% to 46%, and the elongation at break and impact strength increased significantly, but the Tg decreased significantly. N Chikhi et al. [17] toughened EP with amine-terminated butadiene acrylonitrile (ATBN) and showed that by adding only 12.5 phr of ATBN, the impact strength of EP increased by 3 times and the fracture toughness by 1.5 times, while Tg and fracture stress decreased significantly. Wang et al. [18] investigated the mechanical and dielectric properties of HTBN-modified EP. The results showed that with the increase in HTBN content, the glass transition temperature (Tg) and the volume resistivity decrease, while the relative permittivity and dielectric loss tangent increase. The toughness of the composites was enhanced when the HTBN content was more than 15%. The results of the several researchers mentioned above show that even though rubber is effective in toughening EP, there are drawbacks that make the toughened material lose strength, modulus, and heat resistance, which undoubtedly greatly limits its application in toughening EP.

Due to the defects of rubber toughened epoxy systems, since the 1980s, more attention has been turned to the research of toughened epoxy systems using thermoplastic engineering plastics with high heat resistance and good mechanical properties [19,20]. The main thermoplastic resins used for toughening epoxy resins are polyethersulfone, pol-ysulfone, polyetherimide, polyimide, polycarbonate, polyphenylene ether, poly (ether ether ketone) (PEEK), etc. [21,22,23,24,25]. HS Jung et al. [26] toughened EP with PES and showed that the fracture toughness and Tg values of PES-toughened EP increased with the addition of PES, and the tensile and impact strengths of the 10 phr PES-toughened specimens increased by 14% and 106%, respectively. Zhou et al. [27] investigated the effects of phenolphthalein poly (aryl ether ketone) particles (PEK-C) on the mechanical, phase structure, and thermal properties of commercial EP. The results showed that the flexural strength, flexural modulus, and impact strength of the cured EP/PEK-C were increased by 13%, 11%, and 154%, respectively, compared with the pure resin, while the Tg and decomposition temperature were also significantly increased. PEK-C is a high-performance thermoplastic resin with high intermiscibility with epoxy resins and its own advantages of a high glass transition temperature, high modulus, and dimensional stability. According to the previous reports, it is clear that thermoplastic resins could toughen EP without decreasing the strength, modulus, and Tg. However, the toughness can only be effectively achieved when the added amount of thermoplastic resin is large. The thermoplastic resin could form a continuous phase with the epoxy resin spherical domain, or form a co-continuous phase with the epoxy resin. However, the solubility and fluidity of epoxy resin will reduce with the increase in the added amount of thermoplastic resin, which will limit the processing of prepreg.

Although the toughening of EP using single modifiers such as rubber and thermoplastic resins has been studied for decades, the preparation of hybrid ternary composites containing two different modifiers is a relatively new concept. So far, few studies on the synergistic toughening of EP with CTBN and PEK-C have been reported, and it is unknown whether CTBN and PEK-C have synergistic toughening effects on EP and what the synergistic toughening mechanism is. Therefore, in this study, PEK-C and CTBN were added into EP by the hot-melt method, and the toughness, thermal properties, mechanical properties, and phase structures of CTBN/PEK-C-modified EP were investigated. It is hoped to develop a toughening EP system with no reduction in strength and heat resistance, moderate viscosity, and good processing properties. Furthermore, the synergistic toughening mechanism of CTBN/PEK-C-modified EP was studied.

## 2. Experimental

### 2.1. Materials

Diglycidyl ether of bisphenol-A (DGEBA) epoxy resin (E-54) with an epoxy value (Eq/Kg) of 0.51–0.54 and molecular weight of 375.9 was purchased from Jiangsu Nantong Xingchen Synthetic Materials Co., Ltd. (Nantong, China). 4,4-Diamino diphenyl methane (AG-80), epoxy value (Eq/Kg) 0.8–0.87, molecular weight 442, was purchased from Shanghai Huayi Resin Co., Ltd. (Shanghai, China). The curing agent was 4,4-Diaminodiphenyl sulfone (DDS), purchased from Nanjing Chemical Reagent Co., Ltd. (Nanjing, China). Carboxyl-terminated butadiene nitrile liquid rubber (CTBN), grade CTBN1300, Mn = 3150, was supplied by Shenzhen Jiadida New Material Technology Co., Ltd. (Shenzhen, China). Phenolphthalein poly (aryl ether ketone) particles (PEK-C) with a particle size of 60 m, Mn = 36,700~43,000, were provided by Xuzhou Aviation Engineering Plastics Co., Ltd. (Xuzhou, China).

### 2.2. Preparation of Untoughened EP

Firstly, 40 Phr of E-54 and 30 Phr of AG-80 were mixed. After stirring at high speed for 10 min at 100 °C, the temperature was increased to 130 °C, and then 30 Phr of DDS was added. After the DDS was completely dissolved, the resin solution was transferred to a vacuum oven at 120 °C for 30 min, and finally poured into a preheated mold for curing. The curing system is 130 °C/1 h + 160 °C/1 h + 180 °C/2 h + 200 °C/2 h.

### 2.3. Preparation of EP/CTBN Composites

The mixture of 40 Phr of E-54 and 30 Phr of Ag-80 was stirred at 100 °C for 10 min and then heated to 170 °C, and 5 wt % CTBN was added. After 1 h, the mixture was cooled to 130 °C and 30 Phr DDS was added. After the DDS was completely dissolved in the resin, the mixture was transferred to a vacuum oven at 120 °C for degassing, and finally poured into a preheated mold for curing. The curing system is the same as above.

### 2.4. Preparation of EP/PEK-C Composites

The mixture of 40 Phr E-54 and 30 Phr Ag-80 was stirred at 100 °C for 10 min, heated to 170 °C, and then 15 wt % PEK-C was added. After PEK-C was completely dissolved in the resin, it was cooled to 130 °C and 30 Phr DDS was added. After the DDS was completely dissolved, the resin solution was transferred to a vacuum oven at 130 °C for degassing, and finally poured into a preheating mold for curing. The curing system is the same as above.

### 2.5. Preparation of EP/CTBN/PEK-C Composites

Firstly, 40 Phr of E-54 and 30 Phr of Ag-80 were mixed. After high-speed stirring at 100 °C for 10 min, the temperature was raised to 170 °C, and then 5 wt % CTBN was added. After 1 h, 15 wt % PEK-C was added and stirred until PEK-C was completely dissolved in the resin. Then, the temperature was reduced to 130 °C, and 30 Phr DDS was added. After the DDS was completely dissolved, the mixture was transferred to a vacuum oven at 120 °C for degassing. Finally, the mixture was poured into a preheated mold for curing. The curing system is the same as above.

### 2.6. Characterization

A Nicolet 6700 Fourier transform infrared spectrometer was used to analyze the structure of prepared samples. FTIR spectra were recorded on a Bruker’s Tensor II spectrometer in the range of 500 to 4000 cm^−1^ at a resolution of 4 cm^−1^.

According to the GB/T 2567-2008 standard [28], the flexural properties of the samples were carried out by an Instron 3367 universal material testing machine. The specimen size was 80 × 15 × 4 mm^3^, and the number of test samples was no less than 5. According to the GB/T 2571-1995 standard [29], the impact performance of the samples was tested on the PIT501J cantilever impact testing machine. The specimen size was 80 × 10 × 4 mm^3^, and the quoted data are the average of the results from five specimens.

Thermogravimetric analysis (TGA) was carried out under a nitrogen atmosphere with a Q50 thermogravimetric analyzer at a heating rate of 10 °C/min and a heating range of room temperature to 600 °C.

The dynamic mechanical properties of the cured material were determined by using a Q800 dynamic mechanical analyzer (TA Q800, New Castle, DE, USA) in a three-point mode under a nitrogen atmosphere. The test was carried out at a heating rate of 5 °C/min from room temperature to 350 °C with a fixed frequency of 1 Hz.

The rheological properties of each resin system were tested by a DHR-1 type advanced rotational rheometer. The viscosity of the resin was measured at 25 °C~200 °C using the plate viscometer mode. The heating rate was 3 °C/min and the shear rate was 6.28 rad/s.

The fracture morphology of the impact sample was observed by a Japan Hitachi TM300 scanning electron microscope. The fracture surface was sprayed with gold before observation. The PEK-C toughened sample was etched with THF for 72 h before gold spraying.

## 3. Results and Discussion

### 3.1. FT-IR Analysis of Different Toughened Systems

**Figure 1:**
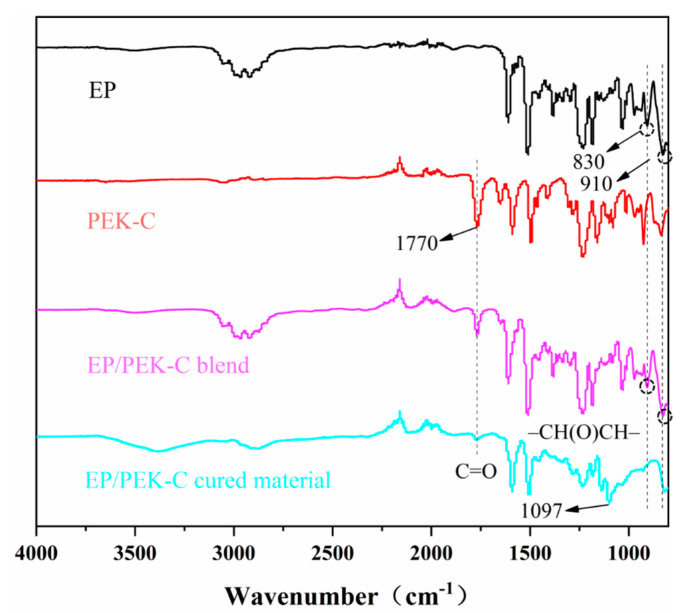
FT−IR spectrum of EP, PEK−C, EP/PEK−C blend, and EP/PEK−C-cured material.

**Scheme 1:**
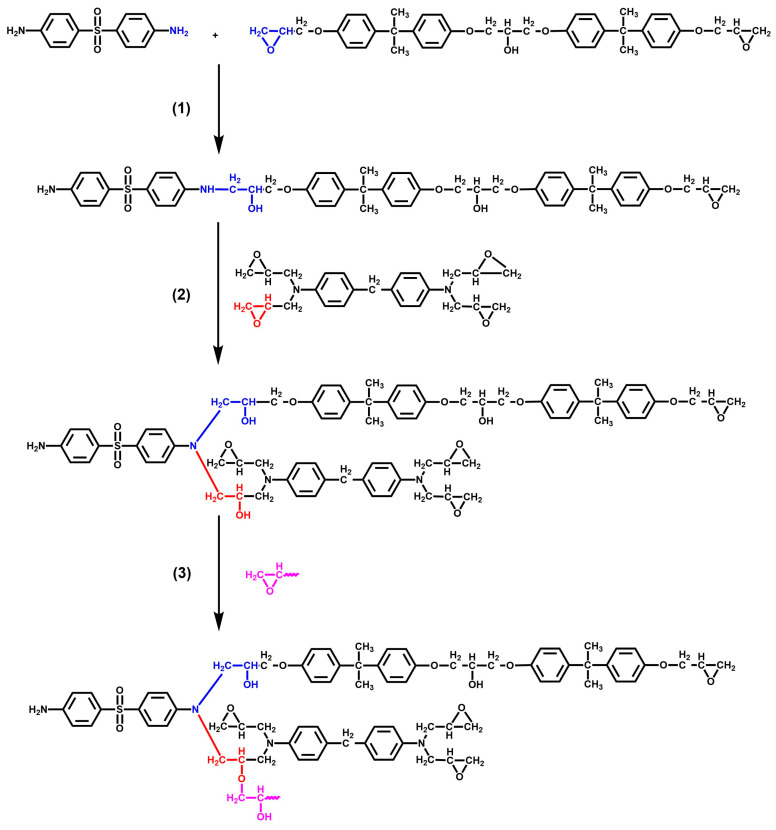
The crosslinking reaction process of EP and DDS.

Figure 1 shows the FT-IR curve of untoughened EP, PEK-C powder, the EP/PEK-C system blend, and EP/PEK-C system-cured material. The peak at 1770 cm^−1^ in the PEK-C FT-IR curve is the C=O stretching vibration absorption peak on PEK-C phenolphthalein. From the FT-IR curve of the EP/PEK-C blends and EP/PEK-C-cured materials, it can be seen that the peak did not disappear. In addition, 3650 cm^−1^ is the characteristic peak of the stretching of the -OH bond on the carboxyl group in pekc, which can form a hydrogen bond with the hydroxyl group on the epoxy matrix. The peak corresponding to the polar hydroxyl group disappears completely in the infrared spectrum after curing, which is due to its participation in the cross-linking reaction of the epoxy system. The carboxyl group on the PEK-C side chain opens the ring and etherifies with the ring-opened epoxy group, eventually forming a cross-linked network. The two peaks at 830 cm^−1^ and 910 cm^−1^ on the FT-IR curve of EP are the characteristic absorption peaks of -CH(O)CH- [30]. The wavenumber and transmittance of the two peaks on the FT-IR curve of the EP/PEK-C blend did not change, but the two peaks on the FT-IR curve of the EP/PEK-C-cured material disappeared. The C-O-C absorption peak was formed at 1097 cm^−1^, indicating that the EP and the curing agent DDS underwent a cross-linking curing reaction, and the reaction was complete. The cross-linking reaction process is as follows: (1) DDS is an aromatic polyamine curing agent, and the active hydrogen atom on the primary amine reacts with the epoxy group in EP to form secondary amines; (2) the active hydrogen in the secondary amine further reacts with the epoxy group to form tertiary amine; (3) the hydroxyl group in the reactant and the epoxy group undergo an etherification reaction, followed by chain expansion, branching, and cross-linking until the formation of a large molecule. The chemical reaction formula of the above three steps is shown in Scheme 1. 

**Figure 2:**
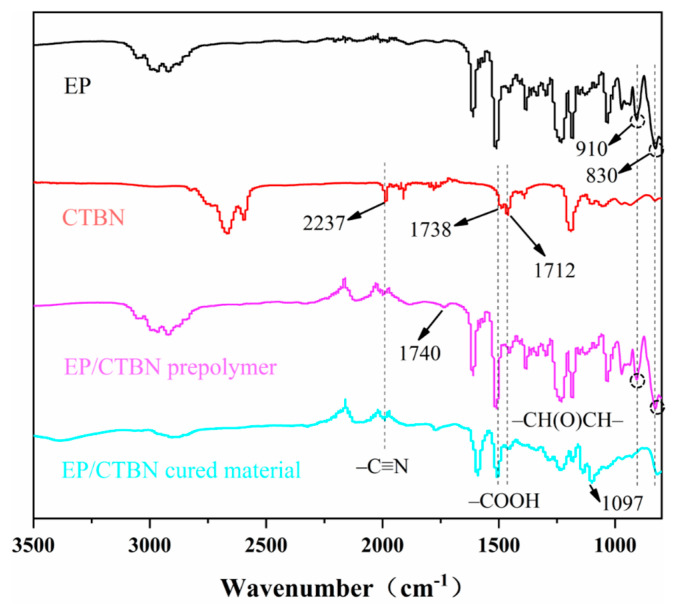
FT−IR spectrum of EP, CTBN, EP/CTBN prepolymer, and EP/CTBN−cured material.

**Scheme 2:**
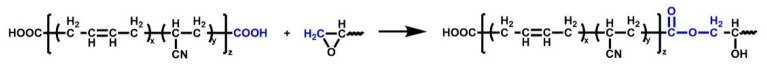
Chemical reaction formula of EP with CTBN.

Figure 2 shows the FT-IR curves of EP, CTBN, EP/CTBN prepolymer, and EP/CTBN-cured material. In Figure 2, the peaks at 910 cm^−1^ and 830 cm^−1^ on the EP FT-IR curve are the characteristic absorption peaks of -CH(O)CH-. The peaks at 1712 cm^−1^ and 1738 cm^−1^ on the CTBN FT-IR curve are the characteristic absorption peaks of -COOH stretching vibration of CTBN [31]. The peak at 2237 cm^−1^ is the characteristic absorption peak of -C≡N stretching vibration. It can be seen from the FT-IR curve of EP/CTBN prepolymer that the two absorption peaks disappeared at 1712 cm^−1^ and 1738 cm^−1^, and a new absorption peak appeared at 1740 cm^−1,^ which is the absorption peak of -COOR stretching vibration. In addition, the two absorption peaks at 910 cm^−1^ and 830 cm^−1^ became weaker, indicating that EP and CTBN underwent an esterification reaction during pre-polymerization [32,33] to generate block polymers and the chemical reaction formula [34] is shown in Scheme 2. As shown in the FT-IR curve of EP/CTBN-cured material, the two absorption peaks at 910 cm^−1^ and 830 cm^−1^ disappeared. The C-O-C characteristic absorption peak was formed at 1097 cm^−1^, which indicated that the EP and the curing agent DDS had a cross-linking curing reaction (etherification reaction), and the reaction was completely. The chemical reaction formula is shown in Scheme 1(3). In addition, in the FT-IR curves of EP/CTBN prepolymer and EP/CTBN-cured material, the -C≡N absorption peak at 2237 cm^−1^ still exists, but the intensity becomes very weak. The reason is that the nitrile group participates in the modification of the network structure during the copolymerization or curing reaction. On the other hand, as the reaction proceeds, the volume fraction of the nitrile group decreases [35].

### 3.2. Analysis of Mechanical Properties

#### 3.2.1. Flexural Properties

**Figure 3:**
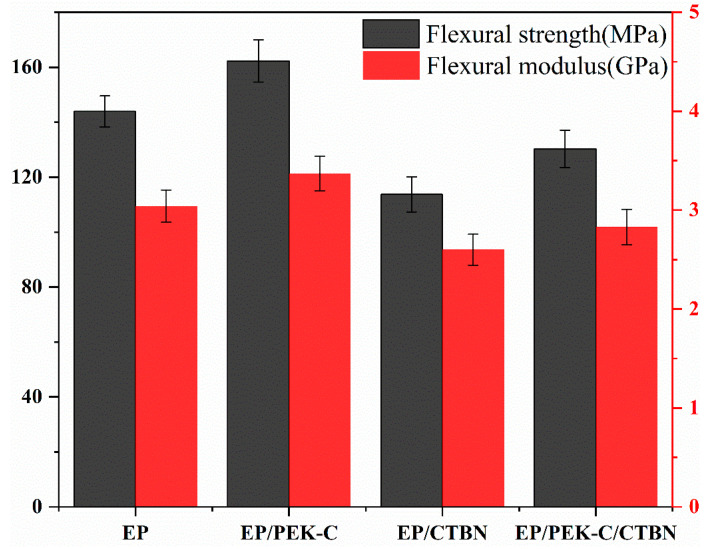
The effects of PEK-C and CTBN on the flexural strength and flexural modulus of EP.

Figure 3 shows the effect of three toughening agents on the flexural strength and flexural modulus of EP. Figure 3 shows the four resin systems’ corresponding flexural strength, flexural modulus, and impact strength values. Among the three toughened systems, the flexural strength and flexural modulus of the EP/CTBN toughening system decreased the most. The flexural strength decreased from 143.93 MPa to 113.75 MPa, a decrease of 21%, and the flexural modulus decreased from 3.04 GPa to 2.6 GPa, a decrease of 15%. Followed by the EP/PEK-C/CTBN synergistic toughening system, the flexural strength decreased from 143.93 MPa to 130.36 MPa, a decrease of 9%. The flexural modulus decreased by 7% from 3.04 GPa to 2.83 GPa. The reason why the flexural strength and flexural modulus of the EP/CTBN toughening system decrease significantly, on the one hand, in the pre-polymerization stage, is because the carboxyl group of CTBN and the EP epoxy group undergo an esterification reaction, so that the cross-linked EP contains a large number of flexible nitrile segments. Although CTBN can cause reaction-induced phase separation during the high-temperature curing process and be separated from the EP/CTBN homogeneous solution, in most cases, the separation of the two phases is incomplete. A small part of CTBN will still be dissolved in the EP continuous phase [36], and the dissolved rubber flexible phase reduces the strength and modulus of EP/CTBN.

The lower decrease in the strength and modulus of the EP/PEK-C/CTBN synergistic system is because the introduction of PEK-C can enhance the entanglement between polymer chains, thereby strengthening the longitudinal and transverse connections between the long chains. During flexural tests, the entanglement between chains inhibits the relative sliding of molecular chains, thereby improving the flexural strength and modulus. Unlike the two toughening systems mentioned above, the flexural strength and flexural modulus of the EP/PEK-C toughening system are higher than those of the untoughened EP system. The flexural strength has increased from 143.93 MPa to 162.31 MPa, an increase of 13%, and the flexural modulus has increased from 3.04 GPa to 3.37 GPa, an increase of 11%. This increase is because the PEK-C molecular chain contains many benzene rings and polar ketone groups. The molecular chain exhibits greater rigidity, making its strength and modulus significantly greater than EP’s and exhibiting an enhanced effect after adding EP. In addition, the presence of many polar groups strengthens the interaction between PEK-C and EP, which positively reinforces the system and improves the strength and modulus of the EP/PEK-C system. In addition, the cured epoxy resin-thermoplastic polymer system has phase segregation and formation of a heterogeneous structure, which also induces performance enhancement.

#### 3.2.2. Impact Performance

**Figure 4:**
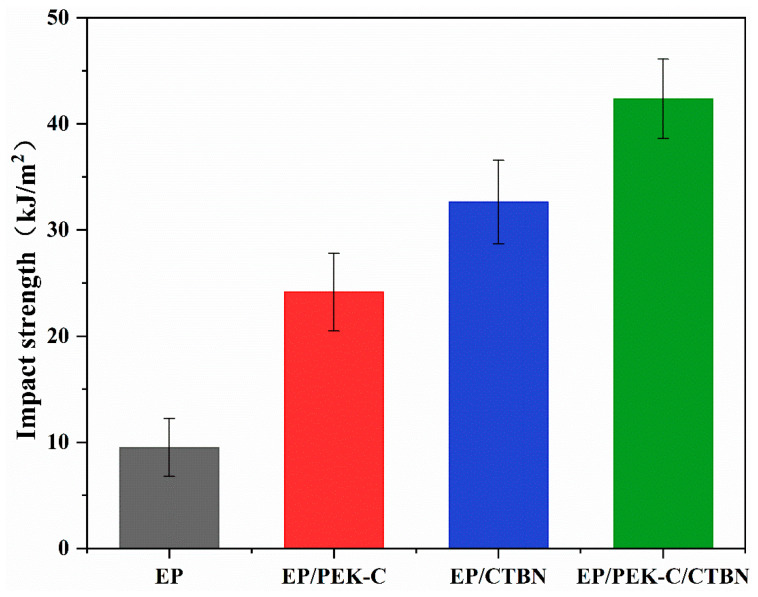
The impact strength value of each toughening system.

Figure 4 shows that the untoughened EP system has the lowest impact strength, indicating the worst toughness. When PEK-C is added, the toughness of the EP/PEK-C system is significantly improved, and the impact strength value increases from 9.51 kJ/m^2^ to 24.16 kJ/m^2^, an increase of 154%. PEK-C undergoes reaction-induced phase separation during the curing reaction and forms a mutually interpenetrating bicontinuous phase structure with EP [37], which causes cracks to inevitably pass through the tough PEK-C continuous phase, thereby triggering the yielding effect of the thermoplastic resin and significantly improving its toughness [38]. The toughness of the EP/CTBN system is further improved compared to the EP/PEK-C system, increasing from 9.51 kJ/m^2^ to 32.64 kJ/m^2^, an increase of 243% compared to the untoughened EP system. The reason for the improvement is that, on the one hand, EP and CTBN undergo esterification reactions to form block copolymers, which introduces a large number of nitrile flexible chain segments into EP [39]. However, the flexible segments will reduce the cross-linking density of the cured EP, reduce the binding of the molecular chain movement, make the matrix prone to plastic deformation, and absorb the fracture energy, thereby improving the toughness of the cured EP.

In contrast, as the curing reaction progresses, the viscosity of the EP/CTBN system gradually increases, inducing phase separation [40]. The rubber particles separate from EP to form a “sea-island” structure, where the sea phase is EP, and the island phase is rubber particles. When the material is damaged, the rubber particles disperse and absorb external impact stress, preventing the propagation of cracks in the EP and slowing down material fracture [41], strengthening and toughening the entire system. Figure 4 shows that the toughness of the EP/PEK-C/CTBN synergistic toughening system is significantly improved compared to the single toughening systems of EP/PEK-C and EP/CTBN, increasing from 9.51 kJ/m^2^ to 42.37 kJ/m^2^, an increase of 342% compared to the untoughened EP system. This suggests that PEK-C/CTBN has a significant synergistic toughening effect on EP related to its unique microstructure. The microstructure and the mechanism for improving toughness are described in detail in the following text.

### 3.3. Analysis of Heat Resistance Performance

**Figure 5:**
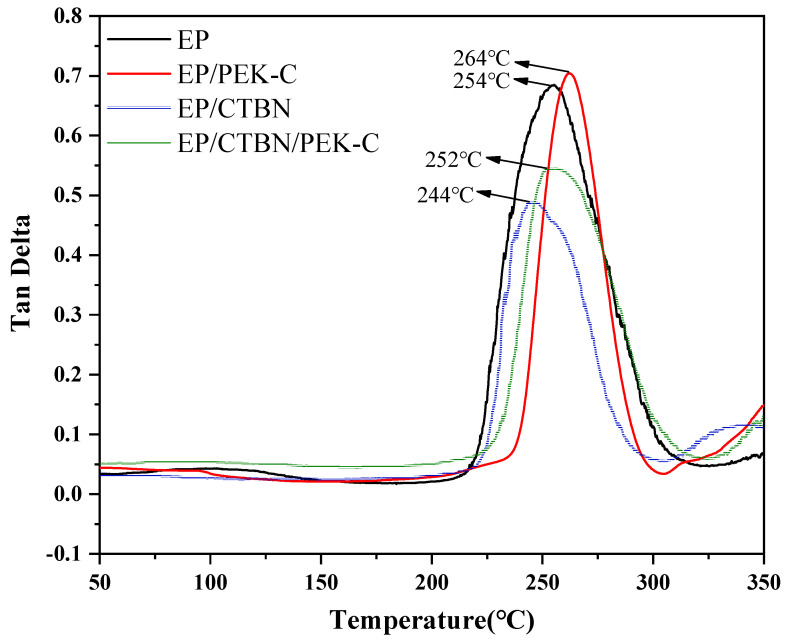
The relationship between modified EP temperature and tanδ.

Figure 5 shows the relationship between the modified EP temperature and tanδ. The graph shows that the addition of PEK-C increases the Tg of EP from 254 °C to 264 °C. The increase is because PEK-C is a macromolecule polymer, and its own Tg is significantly higher than that of EP. Its addition to EP naturally increases the Tg of the whole toughening system. In contrast to the effect of PEK-C addition, the addition of CTBN significantly reduces the Tg of EP from 254 °C to 244 °C. The decrease is due to the flexible chains of the rubber and the rigid chains of EP forming a block copolymer. A portion of the added rubber is not completely separated and dissolved in EP, forming a plastic-to-glass transition [42]. In addition, Figure 5 shows that the Tg of the EP/PEK-C/CTBN toughening system is approximately the same as that of the untoughened EP system, differing only by 2 °C. This is because the Tg increase effect caused by the addition of PEK-C to EP is offset by the decreasing effect caused by the addition of CTBN to EP.

**Figure 6:**
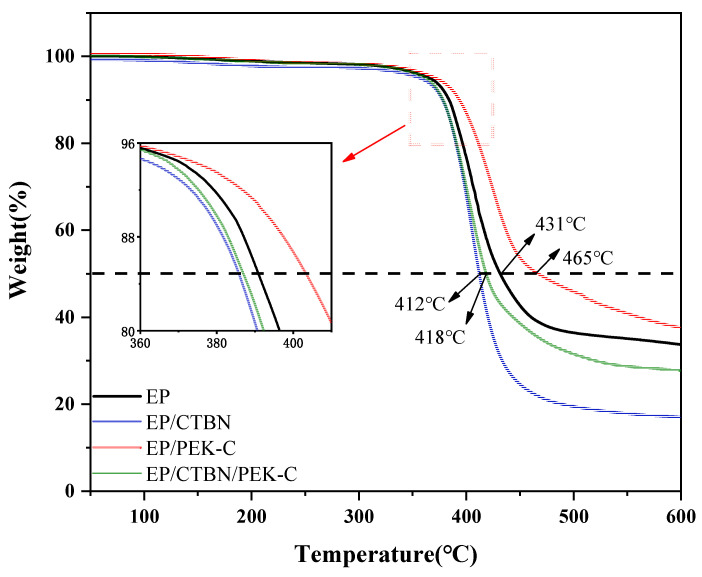
TG curves of different toughening systems.

Figure 6 shows the TG curves of different toughening systems. The graph shows that all four systems begin to lose weight at approximately 136 °C. The temperature between 316 °C and 136 °C is the initial weight loss stage, during which weight loss is relatively slow due to the volatilization of small molecule volatiles. When the temperature interval is between 323 °C and 477 °C, the epoxy resin mainly decomposes and therefore the curve shows a rapid weight loss phase [43]. In this stage, the main chain of the resin casting body undergoes cracking, fusing, and carbonization decomposition, resulting in the maximum weight loss rate. After 478 °C, there is the final weight loss stage, during which the weight loss rate is much lower than that of the previous stage, and the reason for this weight loss should be the decomposition of certain composite resin components.

In addition, the graph shows that the temperatures corresponding to 50% weight loss for the four toughening systems, EP, EP/PEK-C, EP/CTBN, and EP/CTBN/PEK-C, are 431 °C, 465 °C, 412 °C, and 418 °C, respectively. The corresponding weight loss rates at 600 °C are 66%, 62%, 83%, and 72%, respectively. The addition of CTBN reduces the heat resistance of EP, while the addition of PEK-C improves the heat resistance of EP. In addition, the EP/CTBN toughening system begins to lose weight first, and its weight loss rate is the highest in the rapid weight loss stage due to the low molecular weight, short molecular chain, and low cohesive force of CTBN. The reason why the EP/PEK-C toughening system has the lowest weight loss rate in the rapid weight loss stage and the highest carbon residue rate at 600 °C is that the PEK-C molecular chain contains a large number of benzene rings and large phenolphthalein side groups, which greatly increases its own rigidity and heat resistance. After mixing with the EP system, the temperature resistance of the EP/PEK-C system is greatly improved.

Combining the DMA and TG curves leads to the conclusion that adding CTBN to EP decreases its Tg and heat resistance, whereas adding PEK-C to EP increases its Tg and heat resistance. The synergistic toughening of PEK-C/CTBN on EP essentially has no effect on EP’s Tg or heat resistance.

### 3.4. Rheological Properties of Modified Resin

**Figure 7:**
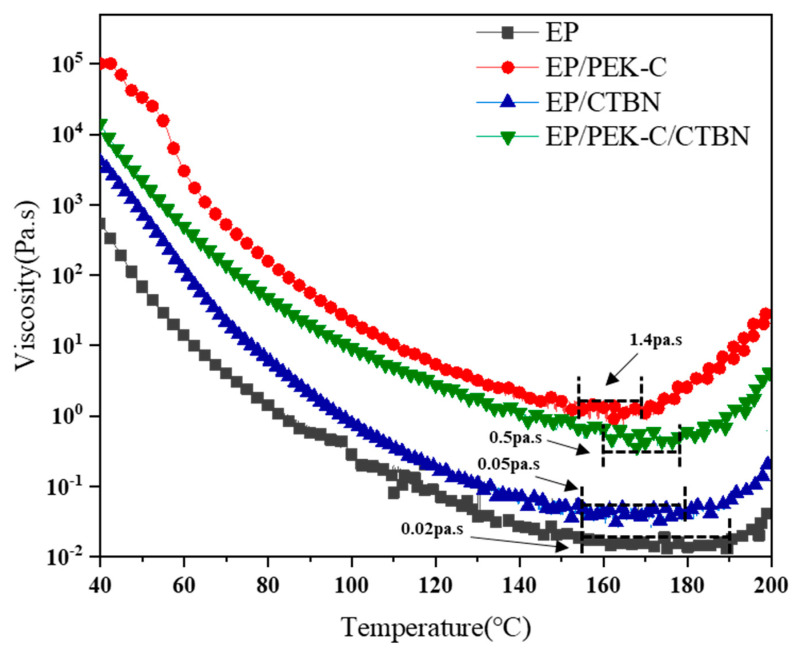
Viscosity−temperature curves of different toughening systems.

The viscosity under different toughening systems significantly impacts the processability of prepreg and composite molding. The dynamic rheological properties of three different toughening systems were investigated in this experiment, and the results are shown in Figure 7.

As shown in the graph, as the temperature rises, the viscosity of all four systems initially decreases, then tends to stabilize, and finally increases again. The initial stage of rapid viscosity reduction is primarily a physical change. As the temperature of the resin increases, the ratio of external heat energy to flow activation energy becomes larger and larger in the process of increasing temperature. The activity and migration ability of the molecular chain segments increase due to thermal activation, and thus the resin viscosity decreases sharply with increasing temperature. After increasing the temperature beyond a certain temperature, the EP and toughening agents are liquid. The viscosity of each blend system reaches a minimum, followed by the appearance of a low-viscosity plateau (the temperature range and viscosity corresponding to the plateau are shown in the marked part of Figure 7). This is because the temperature range has not reached the activation energy temperature required for the curing reaction of the epoxy resin system. The entire resin system has not undergone a curing cross-linking reaction, and its viscosity is at its lowest and most stable. As the temperature rises, the system undergoes a cross-linking reaction and quickly enters the gel state. It then forms a three-dimensional network structure, which limits the movement of the molecules, making the resin show a sharp increase in system viscosity on a macroscopic scale. In addition, the molecular weights of EP, CTBN, and PEK-C increase, with PEK-C having a significantly higher molecular weight than EP and CTBN. The larger the molecular weight, the longer the molecular chain and the higher the likelihood of entanglement. Once entanglement occurs, the flow resistance of the fluid increases and the viscosity increases. Therefore, in Figure 7, the order of gelation occurrence among the four systems is EP/PEK-C > EP/PEK-C/CTBN > EP/CTBN > EP. Figure 7 also shows that in the temperature range of 162.42–181.76 °C, the viscosity of the EP/PEK-C/CTBN synergistic toughening system is significantly higher than that of the untoughened EP system. Still, the viscosity of EP/PEK-C/CTBN is not high. In this low viscosity–temperature range, the preparation of prepreg and the selection of pressure time and other process parameters can be carried out effectively.

### 3.5. Microstructure of Modified Resin System

**Figure 8:**
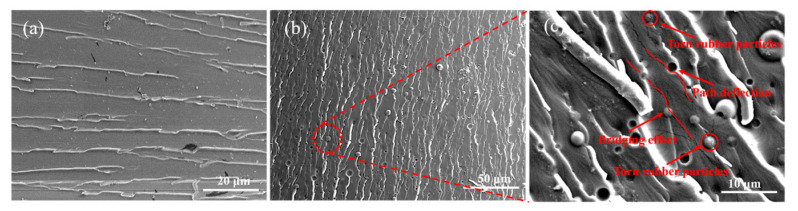
SEM images of (**a**) EP and (**b**,**c**) EP/CTBN toughening system.

After curing, the microstructure determines the resin’s performance under various external forces. Figure 8 shows the SEM fracture surfaces of untoughened EP and EP/CTBN toughening system samples after the impact test. Figure 8a shows that the fracture surface of the untoughened EP sample is smooth and flat, with few branching crack propagation paths and crack lines close to the coastline, exhibiting typical brittle fracture characteristics. The coastline-like morphology of the fracture surface suggests that the resistance encountered during crack propagation is small, and the energy required for crack propagation is low. Therefore, as stated previously, the untoughened EP system has the lowest impact strength and the poorest toughness.

Figure 8b shows that after the addition of CTBN, the fracture surface of the specimen becomes rough, with many folds and sawtooth-shaped cracks exhibiting obvious ductile fracture characteristics. In addition, Figure 8b shows that the rubber particles are uneven and distributed relatively uniformly, with sizes ranging from 1 to 5 μm. During the curing and precipitation process of CTBN, rubber particles that are close together will merge, resulting in larger sizes than those that have not merged. The larger particles can bridge the two sides of the crack (as indicated by the red line in Figure 8c) and prevent it from extending to a highly destructive level. On the other hand, smaller rubber particles induce shear bands through voids, which increases toughness. The uneven sizes of the particles can further enhance the toughening effect and improve the ability to relieve stress concentration [44,45,46]. In addition, Figure 8b shows that the rubber particles leave behind voids when detached from the resin matrix due to stress, resulting in a large surface area that consumes a significant amount of fracture energy. Figure 8c (the two red circles marked) shows that the rubber particles undergo stretching and tearing failure due to stress and cause the crack to diverge and turn due to the pinning effect; additionally, some rubber particles experience plastic deformation [47,48] and become irregular spheres under stress. All these changes caused by stress will consume a substantial amount of fracture energy, effectively preventing the crack from extending and reducing the material’s fracture.

**Figure 9:**
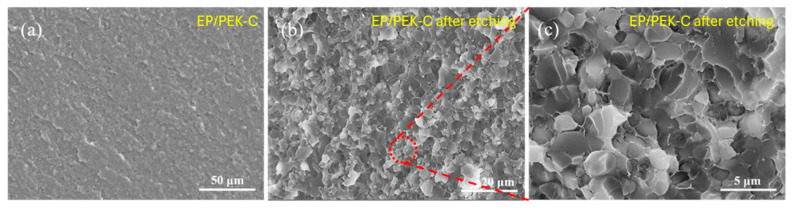
SEM images of EP/PEK-C toughening system before and after etching.

Figure 9 shows the SEM images of the EP/PEK-C toughened system before and after etching with tetrahydrofuran (THF). Figure 9a shows that the fracture surface morphology of the EP after toughening with PEK-C is completely different from that of the untoughened and CTBN-toughened systems. The surface of the fracture is smooth, without coastline or sawtooth-shaped cracks. Figure 9b shows that after THF etching, it can be found that the remaining epoxy-rich component after etching off PEK-C becomes blocky solids of varying sizes, resulting in a no longer smooth fracture surface. In addition, Figure 9c shows that PEK-C and EP actually become a bicontinuous phase structure of EP-coated PEK-C and PEK-C-coated EP, wherein the two phases are interlocked and continuous throughout the entire range, which is exactly the microstructure morphology controlled by the spinodal decomposition mechanism [49]. Compared with the untoughened system, which has only an epoxy-rich extension path, the double continuous phase structure requires the crack to propagate through many PEK-C-rich components. The plastic deformation capacity and energy dissipation of the PEK-C-rich phase are strong. The resistance required to overcome the crack propagation in this phase is also greater, which is why the toughness of the PEK-C toughened system is tougher than that of the untoughened EP system described earlier.

**Figure 10:**
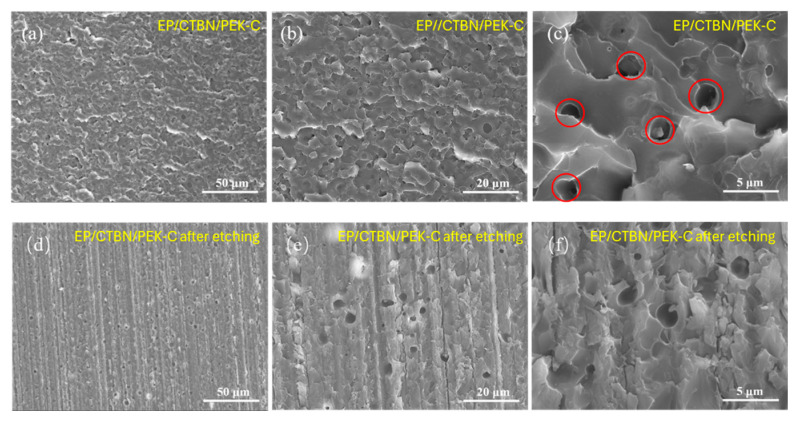
SEM images of EP/CTBN/PEK-C toughening system before and after etching.

Figure 10 shows the SEM images of the EP/CTBN/PEK-C toughening system samples before and after THF etching. In Figure 10a,b, the fracture surface is rough, and there are many damaged surfaces, typical of toughness damage. It is also evident that many microvoids accompany the plastic stretching and cracking phenomena. This is because the rubber particles are subjected to static fluid tension during the curing and cooling process and the action of the triaxial stress field on the crack tip during loading. These two forces superimpose, causing the rupture of holes within the rubber particle or at the interface between the rubber particle and the matrix. The generation of these voids can alleviate the triaxial stress accumulated at the crack tip and on the other hand, will increase the stress concentration on the rubber particles, which makes the cavitation occur further. It also induces the local shear yielding of the matrix around the rubber particles, which in turn leads to the passivation of the crack tip [50], thereby further reducing the stress concentration in the matrix resin and preventing the occurrence of fracture. In addition, multiple stress-whitened regions can be observed in Figure 10a–c. The whitening of the stress is due to the scattering of visible light from the scattering center layer. In this case, it is void of the scattering center due to the cavitation of the CTBN particles [51]. This further proves that the shear yield induced by rubber cavitation is an important toughening mechanism of the EP/PEK-C/CTBN toughening system.

Figure 10c shows that the fracture surface contains a large number of rubber particles (red circle mark) and many bow-shaped cracks of varying thickness, primarily around or through the rubber particles. This is because the rubber particles connect the two sides of the crack through bridging, which constrains and closes the crack propagation. In addition, the particle bridging not only restrains and limits the advancement of the crack leading edge, but the distributed bridging force also anchors the crack at the bridging point, giving the crack leading edge a wavy bow shape.

Figure 10d,e show that the microstructure of the EP/CTBN component left after removing the PEK-C is completely different from the morphology when either PEK-C or CTBN alone is toughened. No cracks propagating in multiple directions are visible in the two images—only linear cracks passing through rubber particles. Figure 10f shows that after the crack passes through the rubber particle, the rubber particle undergoes plastic deformation, transforming into an irregular sphere with numerous broken lumpy bumps or pits in the EP on both sides of the crack in the direction of crack expansion. The analysis suggests that after the EP/PEK-C/CTBN system cracks under external loading, the thermoplastic resin deforms, causing shear yielding. EP and PEK-C form a dual-phase interlocked co-continuous structure, which is closely linked, and the yielding effect of the thermoplastic resin will inevitably drive the changes in EP, thus leaving behind the deformed EP after etching away the PEK-C.

In addition, compared with the SEM image of CTBN alone, it can be observed that the rubber particles in the EP/PEK-C/CTBN synergistic toughening system are rarely exposed on the fracture surface and are primarily embedded in the thermoplastic/thermosetting resin. This indicates that the rubber particles cannot absorb fracture energy through debonding. Combining the above analysis with the current toughening theory of thermoplastic resins and rubber elastomers on EP, the following factors explain why the synergistic toughening effect of the PEK-C/CTBN system is significantly stronger than the individual toughening effects of PEK-C and CTBN: (1) During the CTBN and EP pre-polymerization stage, -COOH in CTBN and -CH(O)CH- in EP undergo an esterification reaction to form a flexible block polymer that can play a toughening role. (2) When CTBN is blended with the resin, reaction-induced phase separation occurs, and CTBN will precipitate and form a similar “sea-island” structure with the thermoplastic/thermoset resin system. When the system is subjected to external force after curing, the small rubber particles can produce shear bands through voids to improve the system’s toughness. The large particles that undergo phase separation undergo tensile tearing and plastic deformation under stress [52,53,54], absorbing a large amount of fracture energy and slowing down material fracture. At the same time, the existence of a flexible block polymer makes the system less bound when it is deformed by force, which is easy to cause plastic deformation. The combined effect of the factors mentioned earlier increases the toughness of the cured material. (3) When encountering cracks, many dispersed-phase rubber particles will have a bridging effect which will play a restraining and closing role on crack expansion and prevent crack expansion to a highly destructive degree. In addition, the distributed bridging force exerts a pinning effect on the crack at the bridging point. The pinning effect causes the crack to deviate from its main plane and increases the surface area, resulting in a significant increase in the energy required for crack propagation. On the other hand, it also causes the coarser primary crack to branch into multiple finer secondary cracks [55]. The secondary cracks with changed expansion direction will inevitably encounter the thermoplastic PEK-C continuous phase. The toughness-rich PEK-C phase will induce greater cooperative deformation at the crack tip, triggering the yielding effect of the thermoplastic resin. The double continuous phase structure of EP and PEK-C will take advantage of the excellent interfacial adhesion to absorb the crack energy and to shear yield against the crack [56].

## 4. Conclusions

Dynamic rheological performance tests show that the EP/PEK-C/CTBN synergistic toughening system meets the requirements of conventional hot-melt prepreg preparation, and the toughening effect of the EP/PEK-C/CTBN synergistic toughening system is significantly higher than that of the single toughening system. The toughening mechanism combines the crack bridging effect, crack pinning effect, shear yielding, and plastic deformation of the rubber particle holes of a conventional single toughening system so that the synergistic system of thermoplastic resin and rubber particles shows an efficient synergistic energy dissipation effect under impact. According to the SEM image, the microstructure of the EP/PEK-C/CTBN synergistic toughening system is entirely distinct from the microstructures of the PEK-C and CTBN single toughening systems. The unique microstructure makes the thermoplastic resin and rubber particles exhibit efficient energy dissipation when subjected to crack damage, which ensures the synergistic toughening system has significantly higher toughness than the single toughening systems.

## Data Availability

The raw data supporting the conclusions of this article will be made available by the authors on request.

## References

[1] Białkowska A., Bakar M., Kucharczyk W., Zarzyka I. (2023). Hybrid Epoxy Nanocomposites: Improvement in Mechanical Properties and Toughening Mechanisms—A Review. Polymers.

[2] Wang J., Xue Z., Li Y., Li G., Wang Y., Zhong W.-H., Yang X. (2018). Synergistically effects of copolymer and core-shell particles for toughening epoxy. Polymer.

[3] Mansour G., Tsongas K., Tzetzis D. (2016). Investigation of the dynamic mechanical properties of epoxy resins modified with elastomers. Compos. Part B Eng..

[4] Ricciardi M., Papa I., Langella A., Langella T., Lopresto V., Antonucci V. (2018). Mechanical properties of glass fibre composites based on nitrile rubber toughened modified epoxy resin. Compos. Part B Eng..

[5] Thomas R., Durix S., Sinturel C., Omonov T., Goossens S., Groeninckx G., Moldenaers P., Thomas S. (2007). Cure kinetics, morphology and miscibility of modified DGEBA-based epoxy resin—Effects of a liquid rubber inclusion. Polymer.

[6] Gunwant D., Sah P.L., Zaidi M. (2018). Morphology and micromechanics of liquid rubber toughened epoxies. e-Polymers.

[7] Qu C., Zhang X., Wang D., Fan X., Li H., Liu C., Feng H., Wang R., Guo K., Tian Y. (2022). Residual stress and thermal properties of rubber-modified epoxy systems for semiconductor package. J. Appl. Polym. Sci..

[8] Sprenger S. (2020). Nanosilica-toughened epoxy resins. Polymers.

[9] Mi X., Liang N., Xu H., Wu J., Jiang Y., Nie B., Zhang D. (2022). Toughness and mechanism of epoxy resins. Prog. Mater. Sci..

[10] Thomas R., Yumei D., Yuelong H., Le Y., Moldenaers P., Weimin Y., Czigany T., Thomas S. (2008). Miscibility, morphology, thermal, and mechanical properties of a DGEBA based epoxy resin toughened with a liquid rubber. Polymer.

[11] Dong L., Zhou W., Sui X., Wang Z., Cai H., Wu P., Zuo J., Liu X. (2016). A Carboxyl-Terminated Polybutadiene Liquid Rubber Modified Epoxy Resin with Enhanced Toughness and Excellent Electrical Properties. J. Electron. Mater..

[12] Imanaka M., Narita I., Nakamura Y., Hisaka S., Yoshida S., Hara K. (2022). Effect of matrix deformability on the fracture properties of epoxy resins modified with core-shell and cross-linked rubber particles. J. Appl. Polym. Sci..

[13] Ali-Asgari Dehaghi H., Mazinani S., Zaarei D., Kalaee M., Jabari H., Sedaghat N. (2013). Thermal and morphological characteristics of solution blended epoxy/NBR compound. J. Therm. Anal. Calorim..

[14] Ma H., Aravand M.A., Falzon B.G. (2019). Phase morphology and mechanical properties of polyetherimide modified epoxy resins: A comparative study. Polymer.

[15] Szymańska J., Bakar M., Białkowska A., Kostrzewa M. (2018). Study on the adhesive properties of reactive liquid rubber toughened epoxy-clay hybrid nanocomposites. J. Polym. Eng..

[16] Tripathi G., Srivastava D. (2007). Effect of carboxyl-terminated poly(butadiene-co-acrylonitrile) (CTBN) concentration on thermal and mechanical properties of binary blends of diglycidyl ether of bisphenol-A (DGEBA) epoxy resin. Mat. Sci. Eng. A Struct..

[17] Chikhi N., Fellahi S., Bakar M. (2002). Modification of epoxy resin using reactive liquid (ATBN) rubber. Eur. Polym. J..

[18] Wang C., Li H., Zhang H., Wang H., Liu L., Xu Z., Liu P., Peng Z. (2016). Influence of addition of hydroxyl-terminated liquid nitrile rubber on dielectric properties and relaxation behavior of epoxy resin. IEEE Trans. Dielectr. Electr. Insul..

[19] Lee S.-E., Jeong E., Lee M.Y., Lee M.-K., Lee Y.-S. (2016). Improvement of the mechanical and thermal properties of polyethersulfone-modified epoxy composites. J. Ind. Eng. Chem..

[20] Yang G., Zheng B., Yang J.-P., Xu G.-S., Fu S.-Y. (2008). Preparation and cryogenic mechanical properties of epoxy resins modified by poly(ethersulfone). J. Polym. Sci. Part A Polym. Chem..

[21] Chen D., Li J., Yuan Y., Gao C., Cui Y., Li S., Wang H., Peng C., Liu X., Wu Z. (2022). A new strategy to improve the toughness of epoxy thermosets by introducing the thermoplastic epoxy. Polymer.

[22] Ji Y., Zhang Y., Wang P., Li Y., Sui J. (2021). Mechanical and thermal properties of epoxy resins modified by a novel thermoplastic-polyimide. Fiber Polym..

[23] Karthikeyan L., Robert T.M., Mathew D., Suma D.D., Thomas D. (2021). Novel epoxy resin adhesives toughened by functionalized poly (ether ether ketone) s. Int. J. Adhes. Adhes..

[24] Li H., Zhao L., Su K., Feng H., Wang D., Qu C. (2021). A comparative study on the rheological, thermal, and mechanical performance of epoxy resin modified with thermoplastics. J. Adhes. Sci. Technol..

[25] Mathis E., Michon M.-L., Billaud C., Vergelati C., Clarke N., Jestin J., Long D.R. (2022). Controlling the morphology in epoxy/thermoplastic systems. ACS Appl. Polym. Mater..

[26] Jung H.-S., Park Y., Nah C.-W., Lee J.-C., Kim K.-Y., Lee C.S. (2021). Evaluation of the mechanical properties of polyether sulfone-toughened epoxy resin for carbon fiber composites. Fiber Polym..

[27] Zhou S., Chen Z., Tusiime R., Cheng C., Sun Z., Xu L., Liu Y., Jiang M., Zhou J., Zhang H. (2019). Highly improving the mechanical and thermal properties of epoxy resin via blending with polyetherketone cardo. Compos. Commun..

[28] (2008). Test Method for Impact Resistance of Resin Casting Body.

[29] (1995). Test Method for Impact Resistance of Resin Casting Body.

[30] Yin X., Xie Z., Liu Q., Yuan X., Hou X., Zhao J. (2021). Synergistic toughening of epoxy resin by CTBN and CM-β-CD. J. Appl. Polym. Sci..

[31] Chen H., Zhu Z., Patil D., Bajaj D., Verghese N., Jiang Z., Sue H.-J. (2023). Mechanical properties of reactive polyetherimide-modified tetrafunctional epoxy systems. Polymer.

[32] Ramos V.D., da Costa H.M., Soares V.L., Nascimento R.S. (2005). Hybrid composites of epoxy resin modified with carboxyl terminated butadiene acrilonitrile copolymer and fly ash microspheres. Polym. Test..

[33] Tripathi G., Srivastava D. (2008). Studies on the physico-mechanical and thermal characteristics of blends of DGEBA epoxy, 3, 4 epoxy cyclohexylmethyl, 3′, 4′-epoxycylohexane carboxylate and carboxyl terminated butadiene co-acrylonitrile (CTBN). Mat. Sci. Eng. A Struct..

[34] Akbari R., Beheshty M.H., Shervin M. (2013). Toughening of dicyandiamide-cured DGEBA-based epoxy resins by CTBN liquid rubber. Iran. Polym. J..

[35] Nigam V., Setua D.K., Mathur G.N. (1999). Characterization of liquid carboxy terminated copolymer of butadiene acrylonitrile modified epoxy resin. Polym. Eng. Sci..

[36] Januszewski R., Dutkiewicz M., Nowicki M., Szołyga M., Kownacki I. (2021). Synthesis and properties of epoxy resin modified with novel reactive liquid rubber-based systems. Ind. Eng. Chem. Res..

[37] Ding Y., Liu R., Liu H. (2020). Investigation on viscoelastic phase separation of phenolphthalein poly (ether ether ketone)/epoxy blends system. Polym. Polym. Compos..

[38] Mimura K., Ito H., Fujioka H. (2000). Improvement of thermal and mechanical properties by control of morphologies in PES-modified epoxy resins. Polymer.

[39] Son B.T., Trung N.N., Lim D.-G., Shin S., Bae J.-Y. (2012). Improvements in thermal, mechanical, and dielectric properties of epoxy resin by chemical modification with a novel amino-terminated liquid-crystalline copoly (ester amide). React. Funct. Polym..

[40] Liu X.-F., Luo X., Liu B.-W., Zhong H.-Y., Guo D.-M., Yang R., Chen L., Wang Y.-Z. (2019). Toughening epoxy resin using a liquid crystalline elastomer for versatile application. ACS Appl. Polym. Mater..

[41] Tiwari S.N., Agnihotri P.K. (2023). Effect of crumb rubber addition on the deformation and fracture behavior of ductile epoxy matrix. J. Appl. Polym. Sci..

[42] Ozturk A., Kaynak C., Tincer T. (2001). Effects of liquid rubber modification on the behaviour of epoxy resin. Eur. Polym. J..

[43] Ahmad H., Shah A., Afaq S.K., Azad M., Arif S., Siddiqi M., Xie L. (2024). Development and characterization of kevlar and glass fibers reinforced epoxy/vinyl ester hybrid resin composites. Polym. Compos..

[44] Dispenza C., Spadaro G., McGrail P.T. (2005). The influence of the rubber carboxyl functionality on the processability and properties of solid rubber modified epoxy mixtures. Macromol. Chem. Phys..

[45] Gao Z., Yu Y., Xu Y., Li S. (2007). Synthesis and characterization of a liquid crystalline epoxy containing azomethine mesogen for modification of epoxy resin. J. Appl. Polym. Sci..

[46] Morancho J., Salla J. (1999). Relaxation in partially cured samples of an epoxy resin and of the same resin modified with a carboxyl-terminated rubber. Polymer.

[47] Dadfar M., Ghadami F. (2013). Effect of rubber modification on fracture toughness properties of glass reinforced hot cured epoxy composites. Mater. Des..

[48] Vijayan P.P., Pionteck J., Huczko A., Puglia D., Kenny J.M., Thomas S. (2014). Liquid rubber and silicon carbide nanofiber modified epoxy nanocomposites: Volume shrinkage, cure kinetics and properties. Compos. Sci. Technol..

[49] Zhang J., Guo Q., Fox B. (2009). Structural and material properties of a rapidly cured thermoplastic-toughened epoxy system. J. Appl. Polym. Sci..

[50] Xi J., Yu Z. (2018). Toughening mechanism of rubber reinforced epoxy composites by thermal and microwave curing. J. Appl. Polym. Sci..

[51] Bian X., Tuo R., Yang W., Zhang Y., Xie Q., Zha J., Lin J., He S. (2019). Mechanical, thermal, and electrical properties of BN–epoxy composites modified with carboxyl-terminated butadiene nitrile liquid rubber. Polymers.

[52] Ocando C., Tercjak A., Mondragon I. (2010). Nanostructured systems based on SBS epoxidized triblock copolymers and well-dispersed alumina/epoxy matrix composites. Compos. Sci. Technol..

[53] Ocando C., Tercjak A., Serrano E., Ramos J.A., Corona-Galván S., Parellada M.D., Fernández-Berridi M.J., Mondragon I. (2008). Micro- and macrophase separation of thermosetting systems modified with epoxidized styrene-*block*-butadiene-*block*-styrene linear triblock copolymers and their influence on final mechanical properties. Polym. Int..

[54] Pearson R.A., Yee A.F. (1991). Influence of particle size and particle size distribution on toughening mechanisms in rubber-modified epoxies. J. Mater. Sci..

[55] Klingler A., Bajpai A., Wetzel B. (2018). The effect of block copolymer and core-shell rubber hybrid toughening on morphology and fracture of epoxy-based fibre reinforced composites. Eng. Fract. Mech..

[56] Gilbert A.H., Bucknall C.B. (1991). Epoxy resin toughened with thermoplastic. Makromol. Chemie. Macromol. Symp..

